# A Potential Benefit of Albinism in *Astyanax* Cavefish: Downregulation of the *oca2* Gene Increases Tyrosine and Catecholamine Levels as an Alternative to Melanin Synthesis

**DOI:** 10.1371/journal.pone.0080823

**Published:** 2013-11-25

**Authors:** Helena Bilandžija, Li Ma, Amy Parkhurst, William R. Jeffery

**Affiliations:** 1 Department of Molecular Biology, Ruđer Bošković Institute, Zagreb, Croatia; 2 Department of Biology, University of Maryland, Maryland, United States of America; Laboratoire Arago, France

## Abstract

Albinism, the loss of melanin pigmentation, has evolved in a diverse variety of cave animals but the responsible evolutionary mechanisms are unknown. In *Astyanax mexicanus*, which has a pigmented surface dwelling form (surface fish) and several albino cave-dwelling forms (cavefish), albinism is caused by loss of function mutations in the *oca2* gene, which operates during the first step of the melanin synthesis pathway. In addition to albinism, cavefish have evolved differences in behavior, including feeding and sleep, which are under the control of the catecholamine system. The catecholamine and melanin synthesis pathways diverge after beginning with the same substrate, L-tyrosine. Here we describe a novel relationship between the catecholamine and melanin synthesis pathways in *Astyanax*. Our results show significant increases in L-tyrosine, dopamine, and norepinephrine in pre-feeding larvae and adult brains of Pachón cavefish relative to surface fish. In addition, norepinephrine is elevated in cavefish adult kidneys, which contain the teleost homologs of catecholamine synthesizing adrenal cells. We further show that the *oca2* gene is expressed during surface fish development but is downregulated in cavefish embryos. A key finding is that knockdown of *oca2* expression in surface fish embryos delays the development of pigmented melanophores and simultaneously increases L-tyrosine and dopamine. We conclude that a potential evolutionary benefit of albinism in *Astyanax* cavefish may be to provide surplus L-tyrosine as a precursor for the elevated catecholamine synthesis pathway, which could be important for adaptation to the challenging cave environment.

## Introduction

Melanin pigmentation protects animals from damage by ultraviolet light and plays important roles in vision, sexual display, mimicry, camouflage, and innate immunity [1,2]. Therefore, albino animals are expected to show reduced fitness, which probably accounts for the low frequency of this trait in most natural populations. In dark caves, however, selection for pigmentation is relaxed, resulting in a myriad of colorless species [3–6]. Thus, along with the reduction or loss of eyes and vision, albinism is one of the hallmarks of troglomorphic animals, which spend their entire life in caves. Albinism is found in a diverse array of cave animals, including planaria, annelids, molluscs, arthropods, and vertebrates [5], but little is known about the evolutionary mechanisms responsible for their colorless phenotypes. Although the benefits of melanization are clear, those conferred by albinism, if any, remain to be elucidated.

The cave animal in which albinism has been most extensively studied is the teleost *Astyanax mexicanus*, which has a pigmented surface-dwelling form (surface fish) and numerous cave-dwelling forms (cavefish) [7–9]. There are 29 different *Astyanax* cavefish populations in the Sierra de El Abra region of northeastern Mexico [10] in which troglomorphic phenotypes have evolved independently several different times [11–13]. Some of these cavefish populations have reduced numbers of pigmented melanophores and can be considered partial albinos, whereas others have no melanophores and are true albinos. Melanin pigmentation can be rescued by exogenous L-DOPA, but not L-tyrosine, showing that melanin synthesis is blocked at its first step in albino cavefish [14].

**Figure 1:**
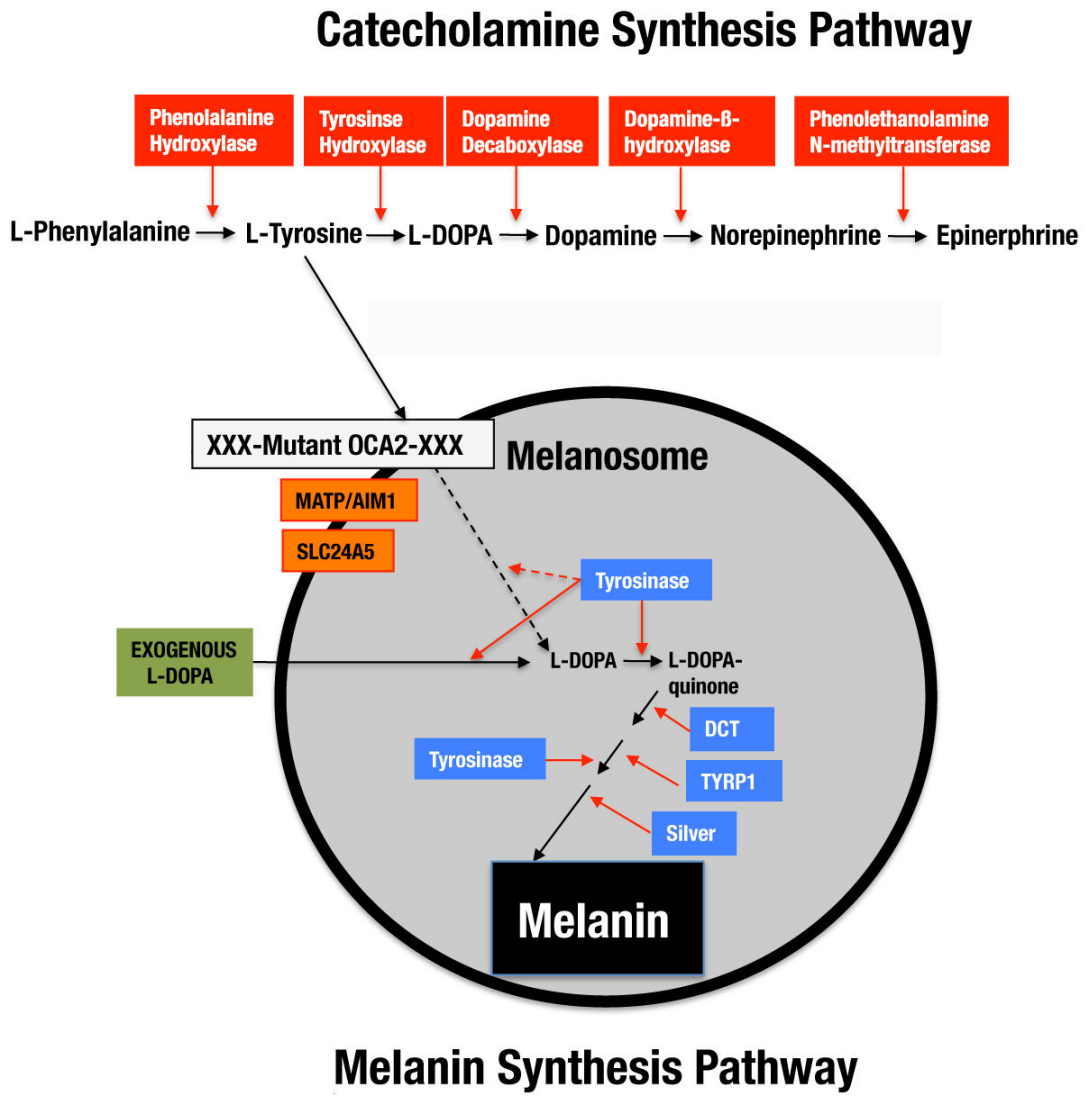
The relationship between the catecholamine and melanin synthesis pathways in *Astyanax* cavefish. The combined pathways begin with the essential amino acid L-phenylalanine, which is converted to L-tyrosine by phenylalanine hydroxylase. L-tyrosine is then converted to L-DOPA either in the catechoamine synthesis pathway (above) or the melanin synthesis pathway (below). The melanin synthesis pathway begins after transport of L-tyrosine into the melanosome (gray sphere) and involves several enzymes (blue boxes) and other gene products (orange boxes) coding for putative transporter proteins essential for melanin synthesis. In albino cavefish, a mutated *oca2* gene (white box with XXX) affects the first step of the pathway prior to tyrosinase function and prevents melanin synthesis. The defect caused by *oca2* loss of function can be rescued by exogenous L-DOPA (green box) [14]. Solid lines: steps that occur in surface fish and in cavefish after L-DOPA rescue of melanogenesis. Dashed lines: steps that are absent in cavefish.

The *oculocutaneous albinism* (*oca2*), *matp/aim1*, and *slc24a5* genes function at the first step of the melanin synthesis pathway (Figure 1 bottom) and presumably make L-tyrosine available for conversion to L-DOPA by tyrosinase. Melanin synthesis proceeds further through a series of well-known reactions [15]. In albino cavefish, mutations in *oca2*, the homologue of the mouse *pink eyed dilution* or *p* gene [16], are the cause of albinism [17]. The *oca2* gene encodes a putative 12-pass membrane protein of unresolved function, although it has been suggested to control L-tyrosine transport [18], melanosome pH [19], or tyrosinase processing [20]. The critical *oca2* mutations are large exon deletions in Pachón, Molino, and Rio Subterraneo (Micos) cavefish, whereas the defect is presumably in a regulatory region in Japones cavefish [17,21]. 

Because *Astyanax* surface fish and cavefish are capable of interbreeding and producing viable hybrids (see 22 for review), they have been used in genetic analysis to explore whether melanophores regress by natural selection, neutral mutation, or both processes. The reduction of cavefish melanophores is a complex genetic trait controlled by a large number of genes, including the *melanocortin-1 receptor* gene [23] as well as *oca2* [17]. The quantitative trait loci (QTL) corresponding to melanophore regression in the F2 progeny of a surface fish x cavefish cross include those of positive and negative polarities, suggesting that random genetic drift may be responsible for this trait [24]. However, the evolutionary cause(s) of albinism, which is itself controlled by the single *oca2* gene with a QTL of negative polarity [17,25], is still uncertain. An important clue may be that melanin synthesis is interrupted at its first step in several different *Astyanax* cavefish populations [14], in unrelated albino cavefish species [26], and also in albino cave planthoppers [27], suggesting a possible convergence in the albinism phenotype at the molecular level. 

There are several possible explanations for why albinism is repeatedly caused by targeting a gene functioning during the first step of melanin synthesis. First, it might be cost effective to interfere with melanin synthesis at its beginning in order to conserve energy. However, it has been argued that since melanin is synthesized from amino acids, it is unlikely to be very costly [28]. Second, *oca2* might be the only gene in the pathway that does not have pleiotropic effects potentially resulting in reduced fitness or lethality. However, *oca2* expression has been detected in tissues other than melanophores [29], suggesting that it might have multiple functions. Third, *oca2* might be unusually sensitive to mutation and thus a frequent target of change. However, mutations also affect other genes in the pathway with the best example being those responsible for the four different kinds of albinism in humans (OCA 1-4) [30,31]. Fourth, a block at the first step could avoid the accumulation of potentially toxic intermediate metabolites, such as dopaquinone [32]. The final possibility, which is explored in the present investigation, is that disruption at the first step could be beneficial because the initial substrate, L-tyrosine, could be used in the alternative catecholamine (CAT) synthesis pathway, which produces dopamine (DA), norepinephrine (NE), and epinephrine (Figure 1 top). 

In addition to regressive traits, *Astyanax* cavefish have evolved many different constructive traits [5], including the enhancement of non-visual sensory [33,34] and gustatory systems [35,36], changes in the hypothalamus [37], and behavioral changes related to feeding efficiency [38,39], which are likely to be under strong selective pressure in a cave environment. The absence of primary productivity limits food resources in the cave habitat [40]. A decrease in the duration of sleep in cavefish relative to surface fish [41] could also contribute to their enhanced food searching phenotype [42]. Feeding and sleep, among many other key physiological processes, are controlled by the catecholamine (CAT) system [43–45]. Accordingly, it was recently shown that decreased sleep in cavefish is regulated by ß-adrenegric signaling, implicating the CAT norepinephrine (NE) in this process, although no changes in NE circuitry could be demonstrated in the brain [46]. CAT levels can be directly modulated by the supply of L- tyrosine [45], suggesting a possible influence of the melanin synthesis pathway. Could a defect in the first step of melanin synthesis be beneficial in cavefish because it would shunt excess L-tyrosine into the CAT synthesis pathway?

 In this study, we have conducted a quantitative analysis of L-tyrosine and CAT levels in *Astyanax* surface fish and Pachón cavefish. We show that L-tyrosine, dopamine (DA), and NE levels are significantly elevated in cavefish larvae and adult brains relative to their surface fish counterparts. In addition, NE levels were discovered to be higher in the cavefish than in surface fish kidneys, which contain the CAT-synthesizing adrenal cells [47]. Finally, we demonstrate that increases in L-tyrosine and DA occur when the first step of melanin synthesis is interrupted by knockdown of the *oca2* gene in surface fish. The results suggest that blocking melanin synthesis at its first step by *oca2* mutations could enhance the CAT pathway and the accompanying physiological and behavioral changes that occur during adaptation to caves. This may be a benefit conferred on cavefish by the evolution of albinism. 

## Materials and Methods

### Ethics Statement

This study was carried out in strict accordance with the recommendations in the National Institutes of Health Guide for the Care and Use of Laboratory Animals. The protocol was approved by the Committee on the Ethics of Animal Experimentation of the University of Maryland (Permit R 09–58). All efforts were made to minimize suffering.

### Biological Materials

*Astyanax mexicanus* surface fish and cavefish (Pachón) laboratory stocks were originally collected at Balmorhea State Park, TX, USA and Cueva de El Pachón, Tamaulipas, Mexico, respectively. Fish were maintained in the laboratory at 23°C on a 14-hr light and 10-hr dark photoperiod [48]. Embryos were obtained by natural spawning and raised at 25°C. Larvae were fed brine shrimp beginning at 10 dpf, and adults were fed TetraMin Pro flakes (Tetra Holding Inc, Blacksburg, VA, USA). Some surface fish embryos were raised in 400 µM phenylthiourea (PTU; Sigma-Aldrich Chemicals, St. Louis, MO, USA) beginning at 14-17 hours post fertilization (hpf) to inhibit pigmentation prior to *in situ* hybridization. 

### High Performance Liquid Chromatography

Simultaneous determination of L-tyrosine, L-DOPA, DA, and NE levels was done by high performance liquid chromatography (HPLC) with pulse amperometric detection according to a procedure modified from Kumarathasan and Vincent [49]. Replicate samples of surface fish and cavefish larvae and young adults (50 animals in each sample) or dissected brains from 3 surface fish and 3 cavefish of the same size and age were homogenized in ice cold RIPA Buffer (Sigma-Aldrich), centrifuged briefly at 8000 x g (4°C), and the supernatants were removed and stored at -80°C. The samples were treated with a mixture of ice-cold 1 M HCl-acetone-water (40:1:5) to precipitate proteins and centrifuged at 10,000 g for 10 min at 4°C. The supernatants were collected and concentrated to 150 µl by evaporation under N_2_ flow and then run through a 30 kDa molecular cutoff filter, collected, and reconstituted with acidified water. The deproteinization and concentration steps were repeated to completely remove proteins. A 15 µl aliquot of each sample was injected into a Prodigy 5 µm ODS column for separation. A mobile phase of water, methanol, sodium citrate, and sodium acetate buffer (pH 4.0) was pumped through the column at a flow rate of 1.0 ml/min. The compounds were detected using an ICS-3000 ED cell with a carbon electrode at a potential of +0.8 v. The L-tyrosine, L-DOPA and CAT profiles were identified by comparison to standards (Sigma-Aldrich) and quantified from the areas of the peaks.

### Enzyme-linked Immunoabsorbent Assay

CAT concentrations were also determined by enzyme-linked immunoabsorbent assay (ELISA) [50–52] using the 2-CAT Research ELISA Kit (Labor Diagnostika Nord GmbH & Co. KG, Nordhorn, Germany). Whole kidneys were dissected from three different surface fish and cavefish adults of the same size and age. Kidneys as well as surface fish and cavefish larvae were homogenized in 200 µl of ice-cold double distilled water. After quick centrifugation at 4°C, 1 µl aliquots of the kidney homogenate or 10 µl aliquots of the embryo homogenate were loaded into each ELISA well. The ELISA procedure was carried out according to the instructions in the kit. Absorbance at 450 nm and a reference wavelength of 620 nm was read using a microplate reader. The CAT concentrations were determined by fitting a standard curve to a series of CAT standards with known concentrations provided in the kit. The relative CAT concentration per sample was calculated by dividing the determined CAT concentration in the sample by the protein concentration of the same sample. This gave a relative CAT concentration of ng CAT/mg protein for each sample. All measurements were done in triplicate.

### RNA Isolation, cDNA synthesis, and RT-PCR

For RNA isolation surface fish and cavefish embryos were homogenized in 10 volumes of Trizol reagent and the lysates were stored at - 80°C. Total RNA was isolated from the stored lysates using the TRIZOL reagent kit (Life Technologies, Grand Island, NY, USA) according to the protocol supplied in the kit. cDNA synthesis was carried out with 2 µg total RNA using the SuperScript^TM^ III First-Strand Synthesis SuperMix kit with oligo (dT) primers (Life Technologies). 

For semi-quantitative RT-PCR, a 975 base pair (bp) region covering exons 7-15 (thus avoiding the exon 24 deletion in cavefish [17]) of the *Astyanax oca2* coding sequence (GenBank DQ232591) was amplified from 1 µl cDNA template in a 50 µl reaction volume using 5’-TGGAGGGCGTCCCAGCTCAG-3’ (forward) and 5’-GGCTGGCTGGGTTGATGCGA-3’ (reverse) primers and the PCR Master kit (Roche Applied Science, Indianapolis, IN, USA). The PCR cycling conditions were one cycle of initial denaturation at 94°C for 2 min, 32 cycles of denaturation (at 94°C for 30 sec), annealing (at 60°C for 30 sec), elongation (at 72°C for 45 sec), and a final elongation step at 72°C for 10 min. The PCR products were sequenced and confirmed as the expected region of the *Astyanax oca2* gene. As a control for RT-PCR, a 343 bp region of 18S rRNA was amplified using the same PCR conditions as above with 5’-GAGTATGGTTGCAAAGCTGAAA-3’ (forward) and 5’-CCGGACATCTAAGGGCATCA-3’ (reverse) primers.

### In Situ Hybridization

*In situ* hybridization was performed as described previously [53] with some modifications. The oca2 975 bp coding region (see above) was amplified from surface fish cDNA by RT-PCR using the Phusion High-Fidelity PCR Master Mix (New England Biolabs Inc, Ipswich, MA, USA) and 5’-TGGAGGGCGTCCCAGCTCAG-3’ (forward) and 5’-GGCTGGCTGGGTTGATGCGA-3’ (reverse) primers. The cycling conditions were one cycle of initial denaturation at 98°C for 30 sec, and 35 cycles of denaturation (at 98°C for 8 sec), annealing (at 70°C for 20 sec), and elongation (at 72°C for 15 sec), and a final elongation step at 72°C for 10 min. The *oca2* amplicon was cloned into the TOPO TA dual promoter-cloning vector (Life Technologies). After confirmation of the sequence, digoxigenin (DIG) labeled RNA probes were made by *in vitro* transcription using the DIG RNA Labeling Kit (SP6/T7) (Roche). The *oca2* anti-sense probe was linearized with XhoI and transcribed with SP6 polymerase; whereas the *oca2* sense probe was linearized with Hind III and transcribed with T7 polymerase. 

Embryos were fixed with 4% paraformaldehyde (PFA) in phosphate-buffered saline (PBS) overnight at 4°C, de-chorionated with watchmaker’s forceps, dehydrated in methanol, and stored at -20° C. Rehydrated embryos were treated with 10 mg/ml proteinase K in PBS containing 0.1% Tween (PBST) for 5–10 min at room temperature, washed twice with PBST, and post-fixed for 20 min with 4%PFA in PBST. Prehybridization was carried out at 60° C for 4 hr in 50% formamide, 5X saline sodium citrate (SSC), 0.1%Tween-20, 1 mg/ml yeast RNA, and 50 µg/ml heparin (hybridization buffer). The pre-treated embryos were incubated overnight in hybridization buffer at 60° C with 1 µg/ml anti-sense or sense *oca2* RNA probes. After hybridization, the embryos were washed twice at 60°C with 50% formamide/ 2 X SSCT (saline sodium citrate; 0.1%Tween-20) for 30 min each, once at 60°C with 2X SSC (15 min), twice at 60° C with 0.2X SSCT (20 min each), and twice with MABT (150 mM maleic acid, 100mM NaCl, pH7.5, 0.1%Tween-20) at room temperature (5 min each). The embryos were incubated with blocking solution (MABT, 2% blocking reagent) for 4 hr at room temperature and then with anti-digoxigenin Fab-alkaline phosphatase (1:5000; Roche) in blocking solution overnight at 4°C. The embryos were washed once with MABT plus 10% sheep serum at room temperature for 25 min and eight more times (45–60 min each time) with MABT at room temperature. The embryos were then washed with PBST and incubated in BM Purple AP Substrate (Roche) at room temperature in the dark. After the signal had developed, the embryos were rinsed in PBST several times to stop the reaction. Embryos were then cleared through a glycerol series in PBS (30%-50%-80%) prior to light microscopic observation and photography. 

The *in situ* hybridized embryos were embedded in Paraplast and sectioned at 10 µm. The sections were mounted on gelatin-subbed slides, either stained with Eosin or left unstained, and viewed by light microscopy.

### Morpholino-Based oca2 Knockdown

For *oca2* knockdown we used the morpholino (MO) based procedure developed previously for *Astyanax* embryos by Yamamoto et al. [35]. The translational blocking MO 5’CTTGTTCTCCAAATACATCACACCT3’, which corresponds to bp -7 to +18 of the *Astyanax oca2* gene, was designed and provided by Gene Tools Inc (Summerton, OR). The control MO 5’CCTCTTACCTCAGTTACAATTTATA3’, which did not correspond to any *oca2* sequence, was also provided by Gene Tools Inc. In preliminary experiments, 200, 400, or 800 pg of *oca2* or control MOs were injected into 1-4 cell stage surface fish embryos, and melanophore differentiation was followed during subsequent development by the formation of black pigmented cells. The injection of 400 pg *oca2* MO into embryos provided maximal melanophore inhibition with minimal effects on normal development and was used in all subsequent *oca2* knockdown experiments.

### L-DOPA Pigmentation Rescue Assay

The L-DOPA pigmentation rescue assays were done according to McCauley et al. [14]. Embryos were fixed in 5% formalin in PBS for 1 hr at 4°C, washed several times in PBS, incubated with 0.1% L-DOPA (Sigma) buffered at pH 7.4 in a sodium phosphate buffer system for 2-4 hrs at 37°C, and terminated by washing specimens in PBS.

### TH Antibody Staining

Dissected kidneys (see above) were fixed overnight in 4% paraformaldehyde in PBS, embedded in Paraplast, and cut into 16-18 µm sections in their head (anterior-most) regions. The sections were placed on L-lysine coated microscope slides, washed twice for 2 min in PBS, blocked in Superblock for 30 min, rinsed twice in PBS for 10 min, and incubated for 1 hr in tyrosine hydroxylase (TH) monoclonal antibody (clone LNC1, Millipore, Billerica, MA) diluted 1:400 in PBS. After primary antibody staining, the sections were rinsed twice in PBS for 10 min, incubated in rhodamine-labeled goat anti-mouse secondary antibody (Millipore) diluted 1:200 in PBS for 60 min, rinsed twice for 10 min in PBS, and viewed by fluorescence microscopy. All procedures were performed at room temperature. TH cells were quantified by counting stained cells in 10 randomly selected 50 µm^2^ areas of the sections.

## Results

### L-Tyrosine, L-DOPA, and Catecholamine Levels in Surface Fish and Cavefish

**Figure 2:**
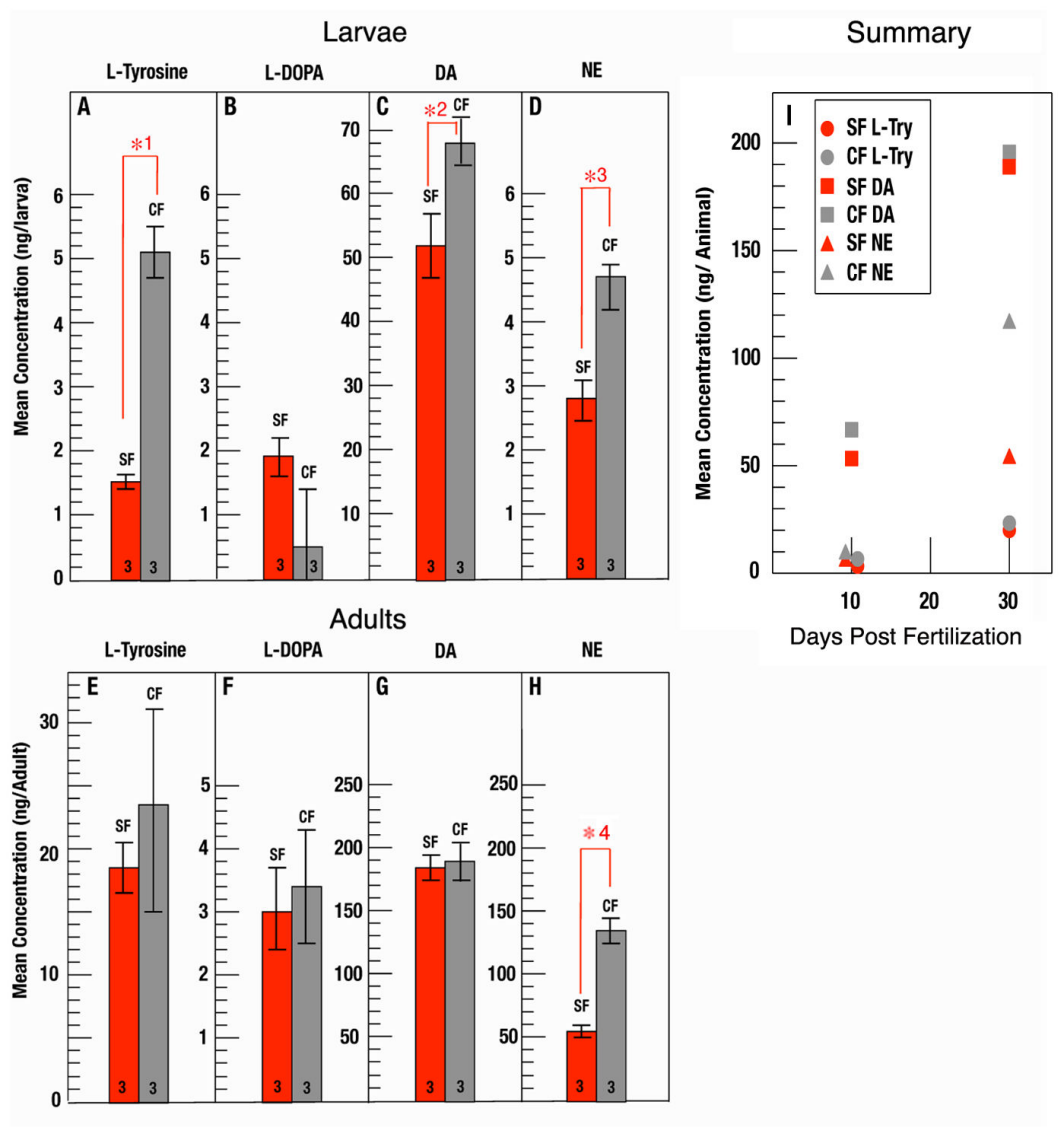
L-tyrosine, L-DOPA and CAT levels in surface fish and cavefish. A-H. L-tyrosine (A, E), L-DOPA (B, F), dopamine (DA; C, G), and norepinephrine (NE; D, H) levels were determined by HPLC at 10 (A-D) and 30 (E-H) dpf in surface fish (red bars) and cavefish (gray bars). The histograms show the mean concentration per larva or adult in lysates (N at base of each bar) each containing 50 animals. Error bars: standard deviation. Red brackets with numbered asterisks indicate significant differences. Statistical analysis was done using Student’s *t* text. *1: p = 0.000. * 2: p = 0.014. *3: p = 0.001, and *4: p = 0.000. Bars without red brackets and asterisks indicate no significant differences. I. Summary of larval and adult L-tyrosine, DA, and NE levels shown on the same scale.

To determine whether changes occur in the CAT synthesis pathway, we simultaneously quantified L-tyrosine, L-DOPA, DA, and NE in samples of developing surface fish and cavefish by HPLC (Figure 2). At 10 days post-fertilization (dpf), synchronously developing surface fish and cavefish embryos have used all of their yolk reserves. At this stage of development, we observed significant differences in L-tyrosine (Figure 2A), DA (Figure 2C), and NE (Figure 2D), but not in L-DOPA (Figure 2B), in whole cavefish larvae relative to their surface fish counterparts. The levels of L-tyrosine were about 4-fold greater and NE was increased almost two-fold in cavefish relative to surface fish larvae. The increase in DA in cavefish was more modest, about 1.5 times that of surface fish. ELISA determination of CAT levels confirmed the elevation of DA in cavefish (see below). The significant differences observed in whole larvae were not apparent in young adults (30 dpf) (Figure 2E-G), with the important exception of NE, which was twice as prevalent in cavefish than in surface fish adults (Figure 2H). However, DA was most abundant of the two CATs in both the larval and adult stages. Figure 2I summarizes the differences detected by HPLC in L-tyrosine, DA, and NE. The results indicate that L-tyrosine and CATs are increased in cavefish larvae and that the enrichment of NE persists and even increases in adults. 

### Catecholamine Increases in the Cavefish Brain and Kidney

**Figure 3:**
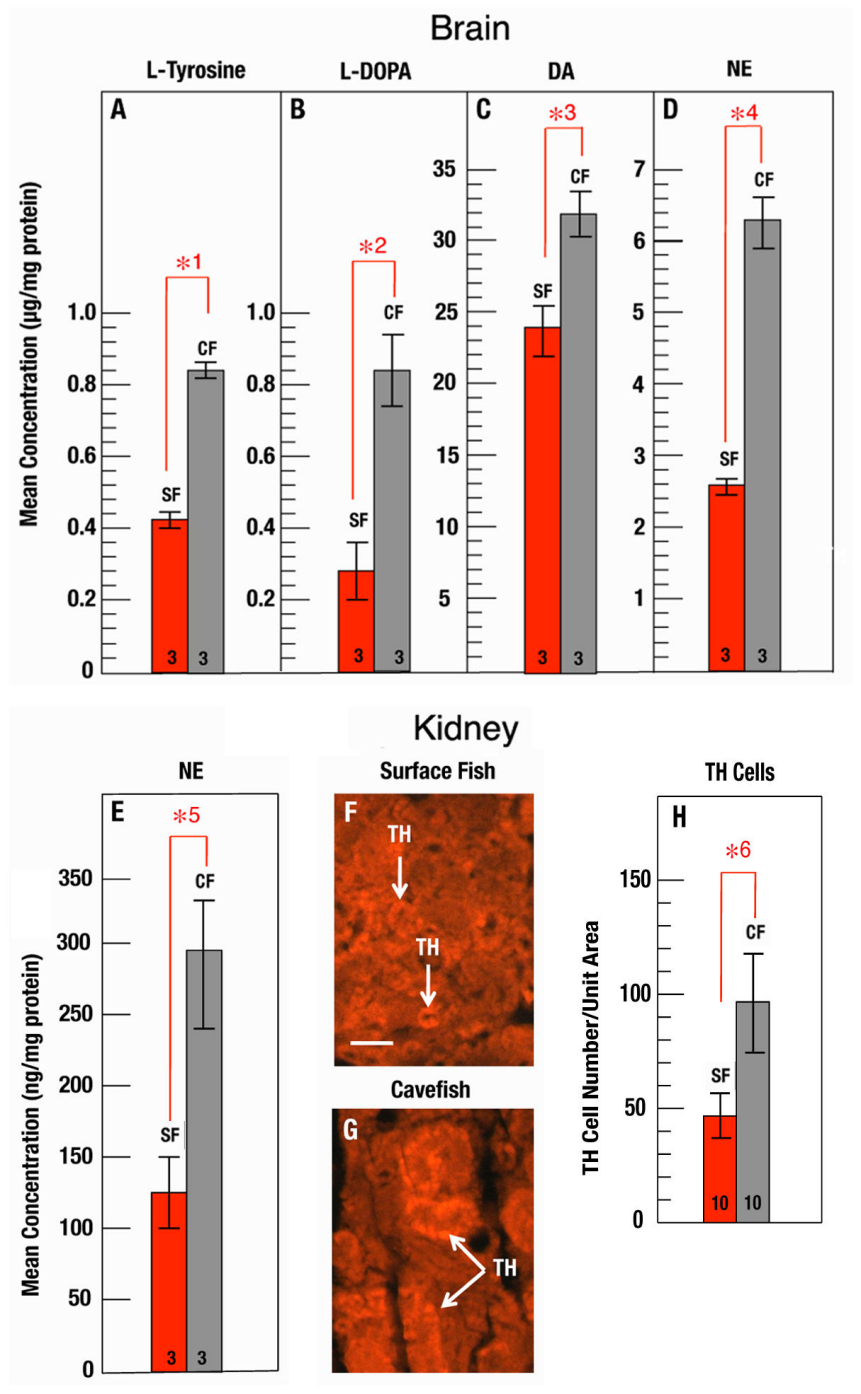
L-Tyrosine, L-DOPA, and CAT levels in adult brains and kidneys. A-D. L-tyrosine (A), L-DOPA (B), DA (C), and NE (D) levels were determined by HPLC of lysates prepared from isolated surface fish (red bars) and cavefish (gray bars) brains. E. NE levels were determined by ELISA of lysates prepared from isolated surface fish (red bar) and cavefish (gray bar) kidneys. Immunostaining of sectioned surface fish (F) and cavefish (G) kidneys with TH antibody shows NE containing cells (arrows) in cavefish and surface fish. Scale bar: 10µm. H. Quantification of TH-staining cells in sections of surface fish (red bar) and cavefish (gray bar) kidneys. N is shown at the base of each bar. Errors bars: standard deviation. Statistical analysis was done using Student’s *t* text with significant differences depicted by red brackets with numbered asterisks. *1: p = 0.000. * 2: p = 0.011. *3: p = 0.021. *4: p = 0.000. *5: p = 0.003, and *6: p = 0.000.

 To further investigate changes in the cavefish CAT pathway, we compared L-tyrosine, L-DOPA, DA, and NE in adult brains and kidneys (Figure 3). First, we measured CAT pathway components in brains dissected from adult (3 month old) surface fish and cavefish by HPLC. As shown in Figure 3A-D, each of the four CAT synthesis pathway components was significantly increased in the cavefish brain. L-tyrosine levels were approximately 2-fold higher, L-DOPA levels about 4-fold higher, and NE levels about 2.5-fold higher in cavefish relative to surface fish brains (Figure 3 A, B, D). As in larvae (Figure 2C), DA showed a more modest increase, and of the two CATs measured, DA was more abundant in the brain (Figure 3C, D). Second, we compared NE levels in dissected surface fish and cavefish kidneys by ELISA and found an approximate three-fold elevation of NE in cavefish kidneys compared to their surface fish counterparts (Figure 3E). Furthermore, in kidney sections stained with TH antibody, which detects cells involved in CAT synthesis, there was an approximate two fold increase in TH-stained chromaffin cells in cavefish compared to surface fish (Figure 3F, G, H). The results indicate that the components of the CAT synthesis pathway are increased in the adult cavefish brain, and that NE is increased in the adrenergic portion of the kidney, suggesting an overall elevation of the CAT synthesis pathway in these organs.

### Oca2 Expression is Downregulated in Cavefish

**Figure 4:**
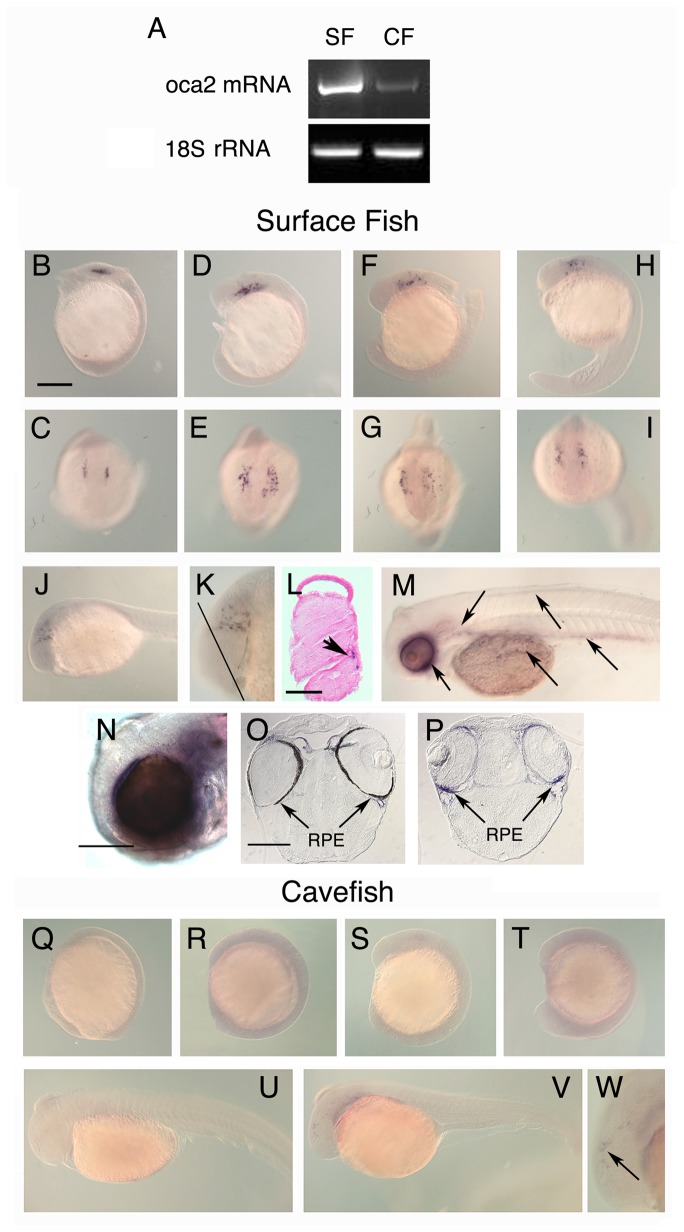
Expression and localization of *oca2* mRNA during early *Astyanax* development. A. Downregulation of *oca2* transcript levels in cavefish (CF) relative to surface fish (SF) at 40 hours post-fertilization determined by RT-PCR amplification of a 975 bp sequence in the middle of the *oca2* coding region. B-W. Localization of *oca2* mRNA in surface fish embryos and larvae (B-P) and *oca2* mRNA downregulation in cavefish embryos and larvae (Q-W) determined by *in* *situ* hybridization. B-I. Surface fish embryos viewed from the lateral (B, D, F, H) and dorsal-rostral (C, E, G, I) sides showing *oca2* expression located bilaterally in cells adjacent to the dorsal midline at 12 (B, C), 13.5 (D, E), 14 (F, G) and 22 (H, I) hpf. J-N. Embryos viewed from the lateral side at 25 (J, K) and 60 (M, N) hpf. Diagonal line in K represents the approximate location of the section shown in L. M. A 60 hpf embryo raised in PTU with arrows showing from left to right *oca2* expression in eyes, otic and pharyngeal regions, yolk sac, trunk dorsal region, and trunk ventral region respectively. N. Higher resolution image of an *oca2* stained 60 hpf embryo showing the pigmented eye. O. Section through a 60 hpf *oca2* stained embryo showing pigmentation in the RPE. P. Section through a 60 hpf PTU treated embryo showing *oca2* staining in the unpigmented RPE. Q-W. Cavefish embryos viewed from a lateral side at 10 (Q), 13 (R), 13.5 (S), 14 (T), 28 (U), and 40 (V, W) hpf. Arrows in L, M, O, P and W show cells expressing *oca2* mRNA. Scale bar in B is 250 µm; magnification is the same in B-J, M, and Q-W. K and W represent 2X magnifications of J and V respectively. Scale bar in L is 125 µm. Scale bars in N and O are 250 µm: magnification is the same in O and P.

To investigate the possible relationship between the enhanced CAT synthesis pathway and the blocked melanin pathway in cavefish, we examined the embryonic expression pattern of the *oca2* gene, whose loss of function has been identified to be the cause of cavefish albinism [17]. We first compared *oca2* expression in surface fish and cavefish embryos at 40 hours of development by semi-quantitative RT-PCR. The results showed strong downregulation of *oca2* expression in cavefish (Figure 4A). Second, we examined the patterns of *oca2* expression during early development in both forms of *Astyanax* by whole mount *in situ* hybridization (Figure 4B-W). Expression of *oca2* was initially detected in surface fish embryos at 12-22 hours post-fertilization (hpf) in loosely organized clusters of cells located on either side of the dorsal midline (Figure 4B-I). Sectioning of *in situ* hybridized embryos showed that these cells were located just beneath the surface (Figure 4L) in the position expected for pre-melanogenic neural crest cells migrating in the dorsal lateral pathway [54]. When the development of pigmentation was inhibited by PTU treatment, *oca2* expressing cells were seen in and around the eyes and the pharyngeal region, the otic vesicle, the yolk sac, and more weakly in the dorsal and ventral regions of the trunk of 60 hpf embryos (Figure 4 M, N). The *oca2* staining was coincident with melanophores in embryos that were not treated with PTU. We also detected *oca2* expression in the retinal pigment epithelium (RPE) of the eye (Figure 4O, P). Cells expressing *oca2* transcripts were generally absent from cavefish embryos, including the body and degenerating eyes, at every developmental stage examined (Figure 4Q-W), although a few weakly stained cells could be detected in the head at about 40 hpf (Figure 4V, W). The downregulation of *oca2* expression in cavefish was not due to the absence of melanoblasts themselves because these cells are detectable by other methods (see below). The results suggest that *oca2* is expressed in the RPE and body melanophores and/or their precursors in surface fish embryos but is downregulated in cavefish embryos. 

### Oca2 Knockdown Inhibits Melanophore Development

**Figure 5:**
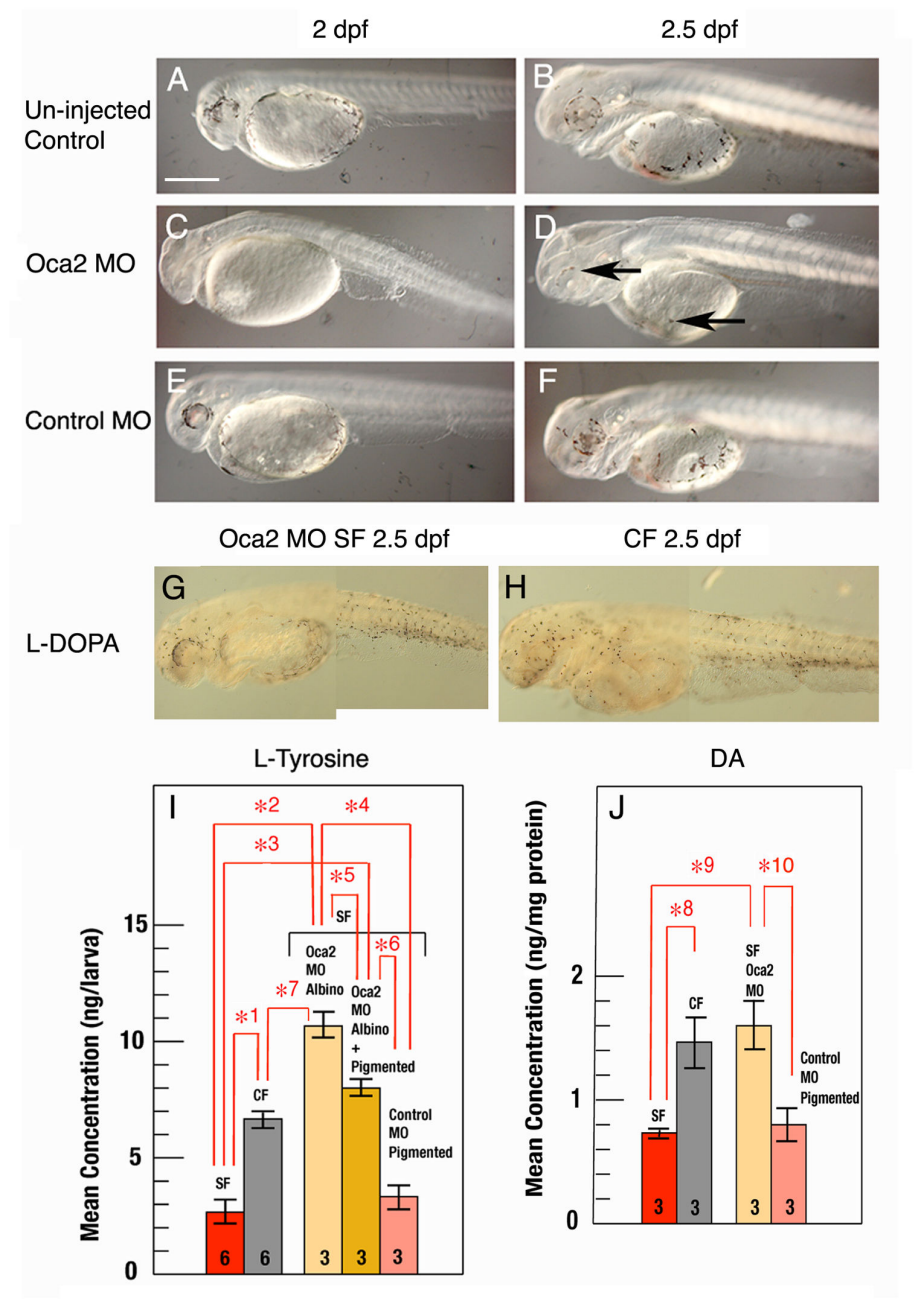
Knockdown of *oca2* affects melanophore development and increases L-tyrosine and DA in surface fish. A-F. The effects of morpholino (MO) mediated *oca2* knockdown on melanophore development in surface fish. A, B. In un-injected embryos eye and body pigmentation is present at 2 and 2.5 dpf. C, D. In embryos injected with 400 pg *oca2* MO, pigmented melanophores are absent at 2 dpf (C) and in most larvae at 2.5 dpf; D shows a morphant larva in which lightly colored melanophores (arrows) have started to appear in a small subset of *oca2* MO injected morphants at 2.5 dpf. E, F. In embryos injected with 400 pg control MO, melanophore development was similar to un-injected embryos. G, H. Composite photographs showing melanin synthesis rescue by L-DOPA in *oca2* morphant embryos (G) and in cavefish at 2.5 dpf (H). Scale bar in A is 500 µm; magnification is the same in A-H. I. HPLC analysis of L-tyrosine levels in lysates containing 50 3.5 dpf surface fish larvae (red bar), cavefish larvae (gray bar), pure albino *oca2* morphant larvae (pale yellow bar; *oca2* MO albino), a mixture of albino and a small number of lightly pigmented *oca2* morphant larvae (see D) (gold bar; *oca2* MO albino + pigmented), and pigmented control morphant larvae (pink bar: control MO pigmented). J. ELISA analysis of DA levels in lysates containing 50-100 2.5 dpf surface fish (red bar), cavefish (gray bar), albino *oca2* morphant larvae (pale yellow bar), and pigmented control morphant larvae (pink bar) at 2.5 dpf. N is shown by the numbers at the base of each bar. Error bars: standard deviation. Statistical analysis was done by the One Way ANOVA test. Significant differences are indicated by red brackets with numbered asterisks: *1,*2, *3, *4, *6, *7: p = < 0.001; *5 p < 0.01: *8, *9, *10 p = < 0.05.

To determine the effects of *oca2* knockdown on the melanin and CAT synthesis pathways, we injected *oca2* translation blocking morpholinos (MO) into fertilized surface fish eggs and followed the development of melanophores in *oca2* and control morphant embryos (Figure 5A-F). In un-injected controls and surface fish embryos injected with control MO, melanophores appeared around the eyes and in the yolk sac by 2 dpf and increased in number at 2.5 dpf (Figure 5A, B, E, F). In contrast, pigmented melanophores did not appear in *oca2* morphant embryos at 2 dpf (Figure 5C), and the majority of these embryos also lacked them at 2.5 dpf (Figure 5D). However, a small number (variable in different experiments but less than 25%) of the 2.5-dpf embryos showed lightly pigmented melanophores, presumably because the initial effects of *oca2* MO injected into cleaving embryos declined during later development. There were no visible effects of the knockdown on eye development, other than the absence of pigmentation (Figure 5C, D). Most *oca2* morphants subsequently developed to the adult stage and eventually became fully pigmented (data not shown). The results show that *oca2* gene knockdown affects melanophore development in surface fish during the early larval stages. 

To further elucidate the effects of *oca2* knockdown on the melanin synthesis pathway, albino *oca2* morphants were subjected to the L-DOPA mediated rescue procedure [14]. The results revealed the presence of melanoblasts capable of using L-DOPA to produce melanin in about the same number in *oca2* surface fish morphants as in albino cavefish embryos (Figure 5G, H). The rescue of melanin synthesis by addition of L-DOPA to *oca2* morphants indicates that the melanin synthesis pathway is blocked by *oca2* gene knockdown at the same position as normally occurs in albino cavefish, the conversion of L-tyrosine to L-DOPA [14]. In summary, the results of the *oca2* knockdown experiments demonstrate that *oca2* controls pigmentation by blocking the first step of the melanin synthesis pathway, which occurs just after the bifurcation of the CAT synthesis pathway (Figure 1). 

### Oca2 Knockdown Increases L-Tyrosine and Dopamine Levels

To answer the question of whether the CAT synthesis pathway is affected by *oca2* knockdown in surface fish, we first determined whether L-tyrosine pools were changed using HLPC. In these experiments, *oca2* morphants were collected at 3.5 dpf and separated into two groups of 50 morphants each (1), those entirely lacking pigmentation (albino morphants) and (2) those containing a majority of albino larvae and a small number of lightly pigmented embryos (albino + pigmented *oca2* morphants) (Figure 5C, D). The results were compared to similar HPLC determinations of L-tyrosine levels at 3.5 dpf in un-injected surface fish and cavefish and to surface fish injected with control MO (pigmented control morphants) (Figure 5I). Cavefish showed an increase in L-tyrosine more than two fold above the levels seen in surface fish. Furthermore, surface fish injected with control MO did not show a significant increase in L-tyrosine relative to un-injected surface fish controls. However, both the *oca2* albino and albino + pigmented morphants showed significant increases in L-tyrosine concentration relative to the surface fish and control morphants, even exceeding that of the cavefish controls. In addition, L-tyrosine was significantly higher in the albino *oca2* morphants than in the albino + pigmented *oca2* morphants, suggesting that pigment formation reduced L-tyrosine in lightly pigmented morphants. Together these experiments show that *oca2* knockdown increases the level of L-tyrosine, and suggests that the expanded L-tyrosine pool may be available as a precursor to the CAT synthesis pathway.

To determine whether *oca2* knockdown has effects on CAT levels, we investigated changes in DA. Since DA concentrations were below the detection levels of HPLC in early larvae, we measured DA concentrations by ELISA. As shown in Figure 5J, the differences in DA levels between cavefish and surface fish detected by ELISA at 2.5 dpf were similar to those observed by HPLC in larva at 10 dpf (Figure 2B). Furthermore, DA levels of *oca2* morphants, but not control morphants, were increased relative to surface fish, attaining the levels of cavefish controls (Figure 5J). 

 In summary, the results of these experiments show that *oca2* knockdown enhances both L-tyrosine and DA in surface fish to similar levels as seen in cavefish, suggesting that mutations in this gene could explain the enhancement of the CAT synthesis pathway in albino cavefish. 

## Discussion

This investigation has revealed a novel relationship between the *oca2* gene and the relative utilization of L- tyrosine as the initial substrate in the melanin and CAT synthesis pathways. Our results suggest that disruption of melanin synthesis by *oca2* loss of function can provide additional L-tyrosine substrate to enhance the CAT pathway. The role of L-tyrosine as a driver of both pathways is established [45,55,56], but to our knowledge the results described here are the first to provide evidence for a reciprocal relationship between the two pathways, a situation that could be involved in the evolution of cavefish albinism.

### Role of oca2 Expression in Melanophore Development and Melanin Synthesis

Genetic studies have revealed an important role of *oca2* mutations in cavefish albinism [17], but until now the pattern of *oca2* expression during *Astyanax* development has remained unknown. Information on embryonic *oca2* mRNA localization is also lacking in most other vertebrate species, with the exception of Medaka, in which a single stage of development was reported [29]. We found that the *oca2* gene is initially expressed in cells located bilaterally along the dorsal midline and immediately below the epidermis of pre-hatching embryos, which is suggestive of a neural crest origin. Later *oca2* expressing cells were present in the ocular region, the otic vesicle, the pharyngeal area, the yolk sac, and the trunk. It is likely that the *oca2* expressing cells in the body are melanophore precursors, a conclusion supported by the results showing that melanophore differentiation is blocked in *oca2* morphants. We also observed strong *oca2* expression in melanin-synthesizing cells of the RPE, which was not observed in *oca2* morphants. Thus, *oca2* expression occurs in the RPE and other cells that function in melanin synthesis during surface fish development.

We also demonstrated that *oca2* expression is decreased in cavefish embryos. Because the mutation described in Pachón cavefish *oca2* is a deletion in the last exon of the coding region [17], it was surprising that downregulation of this gene could be detected at the transcriptional level. Perhaps the loss of the last exon makes *oca2* mRNA unstable in cavefish. Alternatively, there could be undetected changes in *oca2* regulatory sequences, in addition to the coding region deletion. Relevant to this issue, changes in a regulatory sequence have been predicted to control *oca2* downregulation in the Japones cavefish population [17]. If a mutation is eventually found in the *oca2* regulatory region in Pachón cavefish, then it is possible that this change originally conferred loss of function and the previously described coding region deletion occurred secondarily as a neutral effect.

In *Astyanax* and other vertebrates, it has been assumed that *oca2* functions during the first step of the melanin synthesis pathway [4,17]. However, to our knowledge, no direct evidence was previously obtained to support this hypothesis. Our results indicate that the block in melanin synthesis mediated by *oca2* knockdown can be rescued by the provision of L-DOPA to *oca2* morphants, which therefore appear to have all downstream components necessary for melanin synthesis. This result confirms the position of the *oca2* gene at the head of the melanin synthesis pathway. 

### Evolutionary Changes in the CAT Synthesis Pathway

We report new findings showing evolutionary changes in the CAT synthesis pathway hallmarked by higher levels of DA and NE in albino cavefish. Quantitative determinations of CAT pathway components in larvae and adult brains and kidneys revealed that DA and NE are significantly increased in cavefish compared to surface fish. The DA increase was observed in both pre-feeding larvae and adult brains, whereas the NE increase was found in animals at both stages of development, as well as in isolated adult brains and kidneys, the latter containing CAT synthesizing chromaffin cells [47]. The increase in CATs seen in the present study is also consistent with previously reported changes in the cavefish hypothalamus [37], a site of CAT synthesis and function in the brain. 

The hypothalamus contains the centers that regulate feeding activity and appetite [43]. Therefore, in light of the CAT increase it is important to emphasize that cavefish feed more efficiently than surface fish under conditions that replicate the cave environment [42] and that several different behavioral changes regulating the extent of foraging and feeding have evolved in cavefish [38,39]. Furthermore, probably due to excessive feeding, cavefish accumulate fat reserves, which may be an important adaptation to cover periods of low food intake into cave systems. The evolution of these metabolic and behavioral changes could have had a large impact on the ability of *Astyanax* to adapt to the food-depleted cave environment. 

As another possible adaptation to increased foraging, cavefish have evolved a decrease in sleep compared to surface fish [41]. Sleep duration and arousal is controlled by the NE system in vertebrates [44]. Accordingly, a pharmacological study attributed decreased cavefish sleep to ß–adenergic signaling [46]. However, no changes in the pattern of TH expressing neurons were detected in the cavefish brain, implying that major morphological differences in neural circuitry are probably not involved. Our results bring up the possibility that elevated levels of NE, either acting to enhance neurotransmission, paracrine activities, or both, may be involved in sleep loss in cavefish. In future studies, it will be important to explore the intriguing possibility that some of the behaviors dependent on the CAT system in cavefish can be induced in surface fish albinos produced by *oca2* knockdown.

NE also controls the stress response in vertebrates [57]. There is recent evidence that cavefish may have higher levels of stress than surface fish, based on elevated basal cortisol levels [58]. Many more etiological and physiological functions are attributed to dopaminergic and adrenergic systems, including reproduction, motor control, hormone release, and autonomic processes. NE plays a critical role in modulating both immediate and long-term responses to certain behaviorally relevant stimuli and can induce changes in homeostasis [59]. Considering these examples, it becomes apparent how enhancement of the CAT system is potentially beneficial for colonization and survival in cave habitats. Taken together, our results reveal an evolutionary change in overall CAT levels in cavefish that could have promoted their adaptation to an extreme environment.

### L-Tyrosine as a Driver of Increased CAT Synthesis

A key discovery of this investigation is that increases in L-tyrosine and DA are generated by *oca2* knockdown in surface fish embryos. Since *oca2* knockdown also affected melanin synthesis in the RPE and melanophores, this result supports the possibility that expansion of L-tyrosine pools due to the inhibition of melanin synthesis at its first step is a driver of elevated CAT synthesis in cavefish. Transfer of L-tyrosine between parts of the brain might occur by simple diffusion, whereas excess L-tyrosine produced elsewhere in the body could enter the circulating blood serum and cross the blood-brain barrier via the large neutral amino acid carrier transport system [45,60], where it would be available for use by the CAT synthesis pathway. Our conclusion that L-tyrosine is a driver of CAT synthesis is supported by studies in other vertebrates showing that the rate of CAT synthesis is highly sensitive to local concentrations of L-tyrosine [45]. Alternatively, the amount of L-tyrosine substrate is also known to control melanin synthesis [55,56]. Therefore, we propose a relationship between the two pathways in which CAT synthesis can be enhanced at the expense of melanin synthesis.

### Implications of L-Tyrosine Utilization and CAT Enhancement

Two sources of L-tyrosine are used to provide substrate for the synthesis of CATs, melanin, and other tyrosine-derived metabolites. One source is amino acids in food, and the other is enzymatic hydroxylation of L-phenylalanine, which occurs endogenously. We found that L-tyrosine levels are significantly higher in cavefish pre-feeding larvae than their surface fish counterparts but not in feeding adults. During early larval development, teleost embryos depend solely on their yolk reserves for nutrition, and the source of L-tyrosine normally obtained by food intake is not available. Thus, the strongest evolutionary consequences of ramping up the CAT pathway at the expense of the melanin synthesis may be experienced during the early larval stages. Further, our results show that the CAT synthesis pathway is active and higher levels of DA can be detected at 2.5 dpf, suggesting early physiological functions of the CAT system. It is possible that L-tyrosine pools, which can only be replenished endogenously, may be unsaturated in early *Astyanax* embryos, and that blockage of melanin synthesis at its first step could provide surplus L-tyrosine used to increase CAT levels that may be helpful for survival in the cave environment.

Although an increase in DA could be seen after *oca2* knockdown, we were unable to determine whether NE levels were also increased in the morphants. Therefore, the NE increase in cavefish may or may not be due to *oca2* loss of function, and regulatory events downstream in the CAT pathway, such as up-regulation of the enzymes involved in CAT synthesis [61] or downregulation of enzymes involved in CAT degradation, could also be partly or entirely responsible for the NE enhancement in cavefish. Likewise, it is possible that the increase in NE in the cavefish kidney may or may not be related to *oca2* downregulation.

Our results suggest that the endogenous reservoir of L-tyrosine is expanded in albino *Astyanax* cavefish by blocking melanin pathway at its first step. The relationship observed here between the melanin and CAT synthesis pathways may explain why *oca2* has been repeatedly mutated in multiple albino cavefish populations [17,21], although our present conclusions are restricted to Pachón cavefish. Future investigations of other cavefish populations will be needed to determine whether CAT enhancement is a general result of albinism in *Astyanax* cavefish. However, the proposed relationship between melanin and CAT synthesis would not necessarily require a change in the *oca2* gene in other cavefish populations. All that would be necessary is loss of function in one of the three genes known to act at the first step of melanin synthesis in vertebrates, *matp/aim1*, *slc24a5*, and *oca2*, or in the rate limiting enzyme tyrosinase (Figure 1). Thus, an explanation for why *oca2* has been specifically targeted for change multiple times in albino cavefish might still be required. Perhaps both the frequency of mutation and its position in the melanin pathway act in concert to produce this effect. 

The inverse relationship between the block in melanin synthesis and the enhancement in CAT synthesis may be a new example of “secondary pleiotropy”, a type of pleiotropy in which a single mutated gene in a defined biochemical pathway has multiple phenotypic consequences [62]. The classic example of this type of pleiotropy, phenylketonuria (PKU), involves a change in phenylalanine hydroxylase, the enzyme that converts L-phenylalanine to L-tyrosine (Figure 1) [63]. The human illness results in both mental retardation and lighter hair and skin. Interestingly, PKU and the switch between melanin and CAT synthesis described here occur in the same biochemical pathway, illustrating its sensitivity to evolutionary change. 

### A Potential Evolutionary Benefit of Albinism

One difficulty in accepting natural selection as an evolutionary force generating cavefish albinism has been that the benefits of this phenotype were not obvious. Several possible benefits can be envisioned *a priori*, including the conservation of energy and the prevention of toxic intermediate metabolite accumulation, but no evidence has been obtained to support them. Here we hypothesize a benefit entailing the production of excess L-tyrosine and increased CATs as a consequence of interrupting melanin synthesis at its first step. The enhancement of CAT levels would be a possible target of natural selection due to the importance of these compounds as neurotransmitters and paracrine factors that modulate the behaviors that could render cavefish more successful in their natural environment. 

## Conclusions

This investigation has revealed significant increases in the levels of L-tyrosine, DA and NE in the pre-feeding stages of cavefish relative to surface fish. We have also discovered elevations of L-tyrosine and CAT levels in the cavefish brain and an enrichment of NE in cavefish kidneys, organs of major CAT function and secretion. These results bring up the possibility of an important evolutionary change in the cavefish CAT synthesis pathway. The dependency of L-tyrosine and DA enhancements on *oca2* loss of function and accompanying interruption of the melanin synthesis pathway at its first step, the conversion of L-tyrosine to L-DOPA, was demonstrated by MO based *oca2* knockdown in surface fish. These experiments reveal a potential evolutionary benefit of albinism caused by *oca2* loss of function in cavefish: enhancement of the alternative CAT pathway to promote multiple physiological and/or behavioral functions that are adaptive for survival in the extreme cave environment. 
